# Accuracy of Pancreatic Stone Protein for diagnosis of sepsis in children admitted to pediatric intensive care or high-dependency care: a pilot study

**DOI:** 10.1186/s13052-023-01540-6

**Published:** 2023-10-07

**Authors:** Gabriella Bottari, Mariangela Caruso, Emanuel Paionni, Maia De Luca, Lorenza Romani, Mara Pisani, Annalisa Grandin, Livia Gargiullo, Giorgio Zampini, Chiara Gagliardi, Danilo Alunni Fegatelli, Annarita Vestri, Laura Lancella, Ottavia Porzio, Andrea Onetti Muda, Alberto Villani, Marta Ciofi Degli Atti, Massimiliano Raponi, Corrado Cecchetti

**Affiliations:** 1 Pediatric Intensive Care Unit, Bambino Gesù Children’s Hospital, IRCSS, Piazza Sant’Onofrio 4, 00165 Rome, Italy; 2 Department of Anesthesia and Intensive Care, Catholic University of Rome, Residency School of Anesthesia and Intensive Care, Catholic University, Rome, Italy; 3 Clinical Laboratory Unit, Bambino Gesù Children’s Hospital, IRCCS, Rome, Italy; 4 Infectious Disease Unit, Bambino Gesù Children’s Hospital IRCCS, Rome, Italy; 5 Pediatric Emergency Medicine, Bambino Gesù Children’s Hospital, IRCCS, Rome, Italy; 6 General Pediatric and Infectious Disease Unit, Bambino Gesù Children’s Hospital, IRCSS, Rome, Italy; 7 Department of Public Health and Infectious Diseases, Sapienza University of Rome, 00185 Rome, Italy; 8 Scientific Direction, Bambino Gesù Children’s Hospital, Rome, Italy; 9 General Pediatric and Infectious Disease Unit, Pediatric Emergency Medicine, Bambino Gesù Children’s Hospital, IRCCS, Rome, Italy; 10 Clinical Pathways and Epidemiology Unit-Medical Direction, Bambino Gesù Children’s Hospital, IRCCS, Rome, Italy; 11 Medical Direction, Bambino Gesù Children’s Hospital, IRCCS, Rome, Italy

**Keywords:** Biomarkers, Pediatric Sepsis, Pediatric Septic shock, Procalcitonin, C-Reactive protein

## Abstract

**Background:**

Pancreatic Stone Protein (PSP) is one of the most promising diagnostic and prognostic markers. The aim of the study was to assess the accuracy of PSP, compared to C-Reactive Protein (CRP), and Procalcitonin (PCT) for sepsis diagnosis in pediatric patients. Furthermore, we explored the correlation of PSP levels with sepsis severity and organ failure measured with PELOD-2 score.

**Methods:**

Forty pediatric patients were enrolled following admission to pediatric intensive care, high dependency care or pediatric ward. PSP blood levels were measured in Emergency Department (nanofluidic point-of-care immunoassay; abioSCOPE, Abionic SA, Switzerland) on day 1, 2, 3, 5 and 7 from the onset of the clinical signs and symptoms of sepsis or SIRS. Inclusion criteria were: 1) patient age (1 month to 18 years old), 2) signs and symptoms of SIRS, irrespective of association with organ dysfunction. Exclusion criteria were: 1) hemato-oncological diseases and/or immunodeficiencies, 2) pancreatic diseases.

**Results:**

Septic patients showed higher PSP levels than those with non-infectious systemic inflammation. The optimal cut-off in diagnosis of sepsis for PSP at day 1 was 167 ng/ml resulted in a sensitivity of 59% (95% IC 36%—79%) and a specificity of 83% (95% IC 58%-96%) with an AUC of 0.636 for PSP in comparison to AUC of 0.722 for PCT and 0.503 for C-RP. ROC analysis for outcome (survival versus no survival) has showed AUC 0.814 for PSP; AUC 0.814 for PCT; AUC of 0.657 for C-RP.

**Conclusions:**

PSP could distinguish sepsis from non-infectious systemic inflammation; however, our results need to be confirmed in larger pediatric population.

## Background

Sepsis represents one of the major causes of morbidity and mortality in children, with 1.2 million cases per year [1] and a mortality rate between 4 to 50% [2], with most of deaths occurring during the first 48–72 h from sepsis onset [3]. Diagnostic biomarkers serve to establish the presence or absence of a clinical condition: this is the most common class of biomarkers when considering sepsis, wherein there is substantial interest in developing biomarkers that can distinguish infection from non-infectious systemic inflammation. Traditional microbiological blood cultures or other body fluids’ tests remain the gold standard. While specific, they can lack of sensitivity and there is typically a significant time delay between obtaining cultures and generating actionable data. Furthermore, the availability of biomarkers that provide a reliable and timely estimate of the likelihood of bacterial infection before culture results would obviate the risk of treatment with unnecessary antibiotics [4].

Procalcitonin (PCT) is currently the only Food and Drug Administration approved biomarker for sepsis diagnosis [3, 5]. However, when comparing the diagnostic accuracy of PCT and CRP there is a trade-off between sensitivity and specificity and authors reported different diagnostic performance depending on the clinical context [5].

Among emerging biomarkers, Pancreatic Stone Protein (PSP) is one of the most promising diagnostic and prognostic markers of sepsis. It belongs to the family of lectin-binding proteins secreted by acinar pancreatic cells, beta-insular cells, intestinal Paneth cells and fundic gastric cells. In animal models, a rise of serum PSP occurred in all stressed conditions irrespective of pancreatic damage [6]. A review published in 2018 reported that the sensitivity of PSP was comparable to that of CRP and PCT, and that its specificity was better than that of PCT in discriminating between “infected” and “inflamed” patients, underlining the possibility of a bed-side dosage [7].

The aim of our study was to firstly assess accuracy of PSP in diagnosis of sepsis in pediatric patients admitted to pediatric intensive care, high dependency care or general pediatric ward; secondly, we explored the ability of PSP to measure the severity of illness and to predict the risk of mortality.

## Methods

### Study population

This was a prospective longitudinal pilot study, approved by Ethical Committee of Children Hospital Bambino Gesù (n°1103). Parents or child’s next of kin signed the consent form to participate to the study.

Between July 2021 and November 2021, we enrolled pediatric patients admitted to the Children Hospital Bambino Gesù in two departments (emergency department—6 PICU beds, 12 emergency room beds and the 26 general pediatric ward beds—and infectious disease department—21 beds).

Inclusion criteria were: 1) children aged between > 1 month and < 18 years; 2) signs and symptoms of Systemic Inflammation Response Syndrome (SIRS) associated or not to organ dysfunction defined according the International Pediatric Sepsis Consensus Conference criteria [8]. Exclusion criteria were: 1) pediatric patients affected by hemato-oncological diseases, and/or immunodeficiencies; 2) pediatric patients affected by pancreatic diseases; 3) refused consent by parents. All data were anonymized.

The diagnosis of sepsis was based on SIRS plus proven infection. The proven infection was defined by: 1) a positive culture from a sterile body fluid and/or 2) a rapid molecular identification of microorganisms from sterile body fluids by use of Film Array or T2 Bacteria Panel; and/or imaging suggestive for an infection). Categorization in sepsis, severe sepsis or septic shock was made on the International Pediatric Sepsis Consensus Conference criteria [8].

To measure the severity of the organ dysfunction in children admitted to the Pediatric Intensive care unit PELOD-2 score was adopted [9]. A follow-up of 40 days starting from recruitment was performed to monitor the incidence of mortality, the length of stay (LOS) in PICU and in hospital.

### External adjudication committee

An independent external adjunction committee composed of two infectiologist members of the study team (MDL and LR) retrospectively and independently reviewed case report forms for each patient and determined whether a septic event had occurred during the patients’ stay. The committee members had access to all case report forms including results of all microbiological investigations but were blinded to PSP levels.

### Blood sampling and measurement of plasma biomarkers

We measured the PSP blood levels on days 1, 2, 3, 5 and 7 from the onset of clinical signs and symptoms indicating systemic inflammation in pediatric patients at their first admission in PICU, emergency room or pediatric ward depending on the severity of the clinical picture. PSP levels were determined on 50 uL of K2-EDTA anticoagulated venous whole blood samples with the CE-marked IVD PSP CAPSULE on the point-of-care abioSCOPE® device (Abionic SA, Epalinges, Switzerland). The abioSCOPE® is a portable tabletop system that reads capsules containing nanofluidic biosensors to quantify PSP in 7.5 min. The system is comprised of a compact optical measurement unit, a mechatronic system for capsule handling and positioning, an embedded software including data management, connectivity, an interface for human–machine interaction and to display the results.

At the same time points of PSP measurements, we measured the blood levels of White Blood Cells, C reactive Protein, Procalcitonin and Ferritin and PELOD-2 score. Procalcitonin was determined by electrochemiluminescence immunoassay “ECLIA” (Elecsys BRAHMS PCT, COBAS e801, ROCHE). C-reactive protein was measured by immunoturbidimetric assay (Tina-quant C Reactive Protein IV, COBAS c 702, ROCHE). Ferritin was measured with electrochemiluminescence immunoassay “ECLIA”: (Elecsys Ferritin, COBAS e801, ROCHE). White Blood Cells were determined with ADVIA 2120 Hematology System (Siemens Healthineers).

### Study outcome

The primary outcome of the study was to measure the accuracy of PSP in the diagnosis of sepsis, severe sepsis and septic shock in pediatric patients admitted to PICU or high dependency pediatric units. Secondary outcomes included: a) to measure the accuracy of PSP in diagnosis of sepsis in comparison to C-RP and procalcitonin ones. b) to measure the predictive accuracy of PSP for patients’ survival in comparison to procalcitonin and C-RP; c) to measure the correlation between PSP and PELOD-2 score in children admitted to PICU.

### Statistical analysis

Continuous variables data are expressed as mean and standard deviation, median and Interquartile range (IQR), while counts and frequencies are used for the categorical ones. Comparisons between groups were performed by chi-square test or Mann Whitney test, as appropriate.

The diagnostic performances of PSP, PCR, PCT were assessed by the area under the curve (AUC) plotting receiver operating characteristic (ROC) curves designed to differentiate between the patients with sepsis and the patients without sepsis.in which we also considered the survival time observed with a 40 days follow-up. Optimal cut-off values were selected as the values that maximize the sum of sensitivity and specificity.

We determined the diagnostic accuracy, the sensitivity, the specificity and the predictive values, using the Wilson method to obtain the likelihood ratios with the 95% Confidence interval.

All tests were two-tailed. All *p*-values < 0.05 were considered statistically significant. Analyses were performed using R version 4.0.1 (The R Project for Statistical Computing).

## Results

### Baseline characteristics and PSP levels

The final study population consisted of 40 pediatric patients: in Table 1 are reported the main characteristics of the cohort (age, sex, site of infection, etiology, diagnosis and outcome). 45% of the patients had a noninfectious systemic inflammation (Table 2) and 55% had a diagnosis of sepsis with different severity according to the pediatric sepsis definition [8]. The most common source of infection was the Respiratory tract (8/22 pulmonary 36.4%). In 7/22 (31.8%) patients we have observed a combined etiology. Survival outcome observed with a 40-days follow-up evidenced the following: 34 patients survived and 6 deceased with a median PICU length of stay of days 9 [IQR 3–14,25] and a median hospital length stay of 9.5 [IQR 3,75–16,5]. Days. Table 3 reports the relationship between the infection etiology and the severity of clinical picture in patients with sepsis. The median PELOD-2 score for those patients was 3 (0–5).

**Table 1:** Demographic, infective and prognostic characteristics of the pediatric population observed

Parameter	Survival (*n* = 34)	Non Survival (*n* = 6)	All (*n* = 40)
**Age**			
•Infants	13 (38%)	1 (17%)	14 (35%)
•Pre-scholar	8 (24%)	1 (17%)	9 (22,5%)
•Scholar	4 (12%)	2 (33%)	6 (15%)
•Adolescents	0	2 (33%)	11 (27,5%)
**Sex**			
•Female	18 (53%)	5 (83%)	23 (57,5%)
•Male	16 (47%)	1 (17%)	17 (42,5%)
**Site of infection**			
Blood Stream	1 (3%)	2 (33%)	3 (7,5%)
•Respiratory tract	8 (24%)	0 (0%)	8 (20%)
•Abdomen	1 (3%)	0 (0%)	1 (2,5%)
•Other	3 (9%)	0 (0%)	3 (7,5%)
•Combined	5 (15%)	2 (33%)	7 (17,5%)
•None	16 (47%)	2 (33%)	18 (45%)
**Etiology**			
•Bacterial	5 (15%)	1 (17%)	6 (15%)
•Viral	4 (12%)	1 (17%)	5 (12,5%)
•Combined	9 (26%)	2 (33%)	11 (27,5%)
•None	16 (47%)	2 (33%)	18 (45%)
**Diagnosis**			
•SIRS	9 (26%)	0 (0%)	9 (22,5%)
•SIRS and Organ Dysfunction	7 (21%)	2 (33%)	9 (22,5%)
•Sepsis	6 (18%)	0 (0%)	6 (15%)
•Severe sepsis	9 (26%)	1 (17%)	10 (25%)
•Septic shock	3 (9%)	3 (50%)	6 (15%)
**Length of PICU stay** ***median [Q1-Q3]***	7,5 [2,25 – 13]	14,5 [11–18, 75]	9 [3–14, 25]
**Length of Hospital stay** ***median [Q1-Q3]***	12 [6,25–18,25]	0,5 [0–1]	9,5 [3, 75 - 16, 5]
**PELOD—2 *median [Q1-Q3]***	3 [0–4, 8]	6 [2, 5]	3 [0 – 5]

**Table 2:** Reported causes of systemic inflammation of no infectious etiology

Etiology of SIRS	All = n 18
trauma/politrauma	4 (22%)
cerebral hemorrhage	2 (11%)
solid tumor	1 (5,5%)
inflammatory/reumatologic disease	2 (11%)
respiratory failure in central hypoventilation	1 (5,5%)
chemical/inflammatory pneumonia	3 (17%)
fever of unknow origin	5 (28%)

**Table 3:** The relationship between microbiological and sepsis characteristics of the sepsis patients

Variable	Sepsis *N* = 6 (27%)	Severe sepsis *N* = 10 (45%)	Septic shock *N* = 6 (27%)	Total *N* = 22
Etiology				
Bacterial	4	1	1	6 (27%)
Polymicrobial	0	6	5	11(50%)
Viral	2	3	0	5 (23%)
Focus				
Blood stream	1	0	2	3 (14%)
Pulmonary	2	6	0	8 (36%)
Abdominal	1	0	0	1 (4,5%)
Combined	1	2	4	7 (32%)
Other	1	2	0	3 (14%)

The median concentrations of PSP at different time points were 88,00 ng/mL (day 1) [range 19–601; IQR range 58,50–318.8]; 104,00 ng/mL (day 2) [range 19–601; IQR range 51.7 – 268.2]; 119,00 ng/mL (day 3) [range 19–601; IQR range 54.0–261.0]; 135,00 ng/mL (day 5) [range 19–601; IQR range 66.0–311.0]; 171,50 ng/mL (day 7) [range 19–601; IQR range 47.5–278.5] ng/ml. Median concentrations of PSP in relation to the diagnosis of SIRS of infectious etiology (sepsis) versus SIRS without a noninfectious etiology and to the severity of illness were 140.0 ng/mL (sepsis) [IQR range 58.0- 200.2]; 92,5 ng/mL (severe sepsis) [IQR range 55.2- 156.7]; 454 ng/mL (septic shock) [IQR range 428.8- 483.0]; 62.5 ng/mL (SIRS) [IQR range 27.0- 68.0]; 129 ng/mL (SIRS plus organ dysfunction) [IQR range 111.0- 452.0].

### Relationship between Biomarkers levels and diagnosis of sepsis

**Fig. 1:**
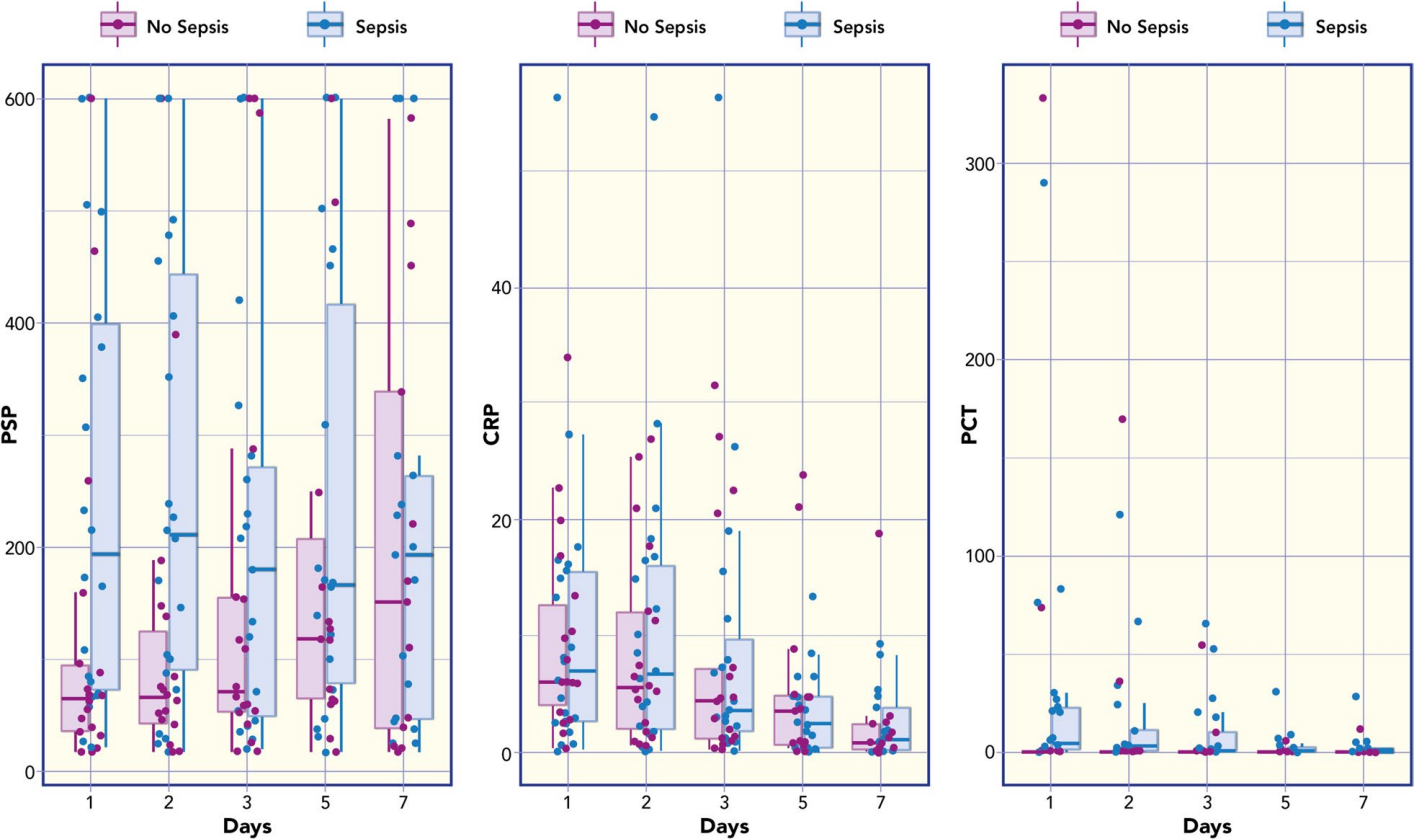
Box plots of Pancreatic Stone Protein (PSP), C-Reactive Protein (CRP) and Procalcitonin (PCT) median value at different time points and in relation to the diagnosis of SIRS versus sepsis

**Fig. 2:**
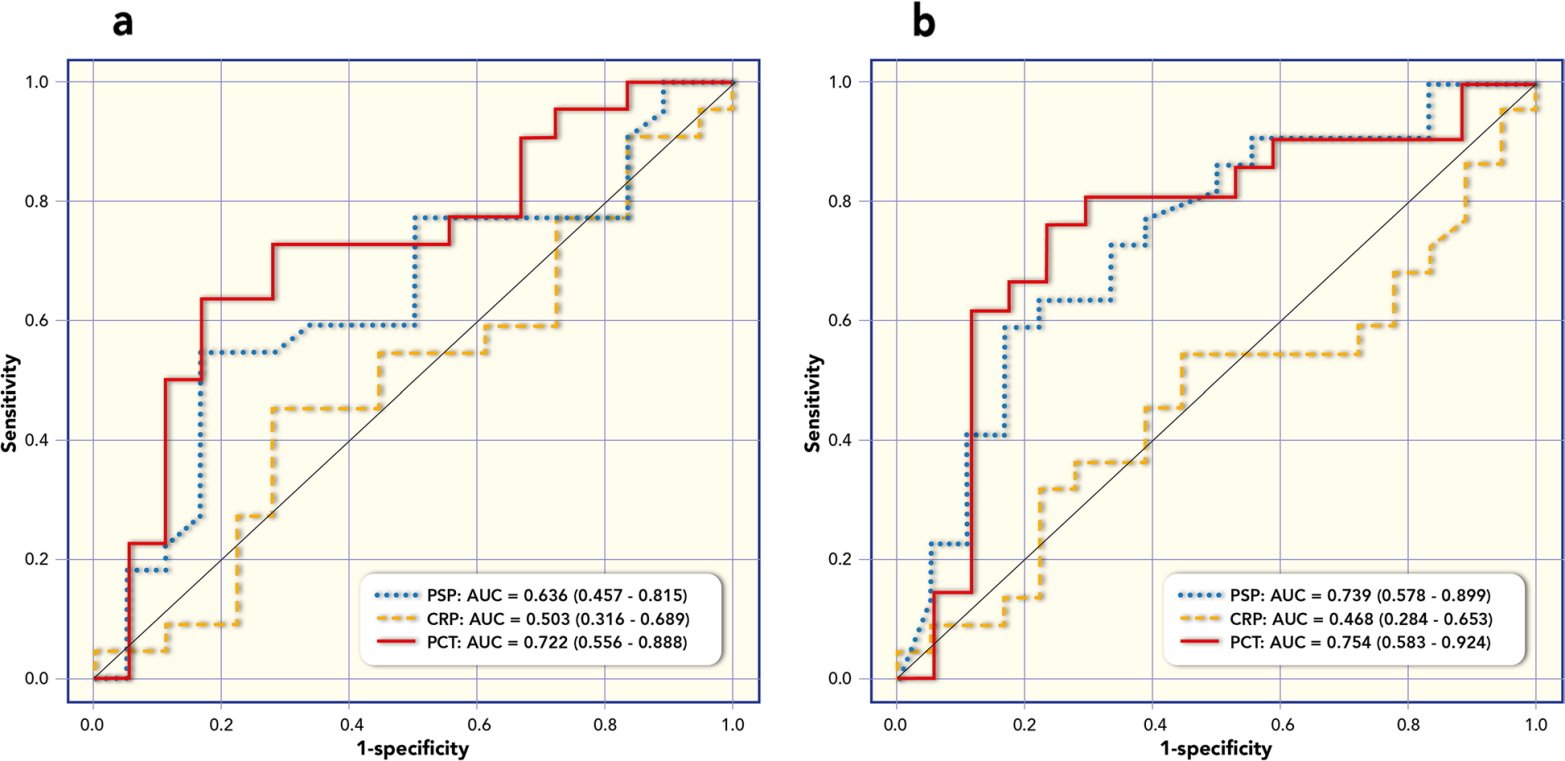
Receiving operating curve (ROC) analysis of Pancreatic Stone Protein (PSP), C-Reactive Protein (CRP) and Procalcitonin (PCT) for diagnosis of sepsis (sepsis versus SIRS). **a** reports Area under the curve (AUC) of median values of biomarkers Pancreatic Stone Protein (PSP), C-Reactive Protein (CRP) and Procalcitonin (PCT) at day 1. **b** reports Area under the curve (AUC) of median values of biomarkers Pancreatic Stone Protein (PSP), C-Reactive Protein (CRP) and Procalcitonin (PCT) at all time points (day 1, 2, 3, 5, 7)

PSP concentration was correlated with PCT (Rho = 0.648; *p* < 0.001); with Ferritin (Rho = 0.350; *p* = 0.184); with White Blood Cells (Rho = 0.139; *p* = 0.491). Figure 1 shows box plots of PSP, CRP and PCT (median values at different time points and in relation to the diagnosis of SIRS versus sepsis). The receiving operating curve (ROC) analysis of the median values at day 1 showed an Area Under the Curve (AUC) of 0.636 for PSP; AUC of 0.722 for PCT; AUC of 0.503 for C-RP (Fig. 2a). Based on ROC analyses, the optimal cut-off in diagnosis of sepsis for PSP at day1 was of 167 ng/ml resulted in in a sensitivity 59% (95% IC 36%—79%) and a specificity of 83% (95% IC 58%-96%).

For all the three biomarkers (PSP, PCT, and C-RP) ROC analysis was performed based on the median values of the measures at different time points. The area under the curve (AUC) was 0.739 for PSP; AUC for PCT was 0.754; AUC for C-RP was 0.468 (Fig. 2b). The corresponding optimal cut-off for PSP in the diagnosis of sepsis was 166 ng/ml, resulting in a sensitivity of 54% (95%CI: 32%—76%) and a specificity of 83% (95%CI: 59%—96%).

Table 4 reports the diagnostic performances of the three biomarkers (day 1 and median times) in distinguishing sepsis fromnon-sepsis in the analyzed population.

**Table 4:** Diagnostic performance of biomarkers including median time of five timepoints (T1 = day 1, T2 = day 2, T3 = day 3, T4 = day 5, T5 = day 7) and the median of timepoint 1 (T1 = day 1) in distinguishing sepsis patients from non sepsis patients

Marker	AUC 95%CI	Cut-off	Sensitivity 95%CI	Specificity 95%CI	PPV 95%CI	NPV 95%CI	LR + 95%CI
PSP Median value	0.636 (0.457–0.815)	166.0	0.54 (0.32, 0.75)	0.83 (0.58, 0.96)	0.80 (0.51, 0.95)	0.60 (0.38, 0.78)	3.7 (1.2, 11.2)
C-RP Median value	0.503 (0.316–0.689)	7.08	0.45 (0.24, 0.67)	0.72 (0.46, 0.90)	0.66 (0.38, 0.88)	0.52 (0.31, 0.72)	2.0 (0.83, 4.8)
PCT Median value	0.722 (0,556–0.888)	1.6	0.63 (0.40, 0.82)	0.83 (0.58, 0.96)	0.82 (0.56, 0.96)	0.65 (0.42, 0.83)	4.67 (1.58, 13.77)
PSP T1	0.739 (0.578–0.899)	167.0	0.59 (0.36, 0.79)	0.83 (0.58, 0.96)	0.81 (0.54, 0.96)	0.62 (0.40 0.81)	2.80 (1.24, 6.30)
C-RP T1	0.468 (0.284–0.653)	6.2	0.54 (0.32, 0.75)	0.55 (0.30, 0.78)	0.60 (0.36, 0.80)	0.50 (0.27, 0.72)	1.50 (0.79, 2.86)
PCT T1	0.754 (0.583–0.924)	1.1	0.71 (0.47, 0.88)	0.76 (0.50, 0.93)	0.78 (0.54, 0.93)	0.68 (0.43, 0.87)	3.20 (1.47, 6.96)

*PSP* Pancreatic Stone Protein, *C-RP* C reactive protein, *PCT* procalcitonin, *AUC* Area Under the Curve, *CI* confince interval, *PPV* positive predictive value, *NPV* negative predictive value, *LR* + positive likelihood ratio

### Relationship between Biomarkers levels and outcome (survival versus no survival)

**Fig. 3:**
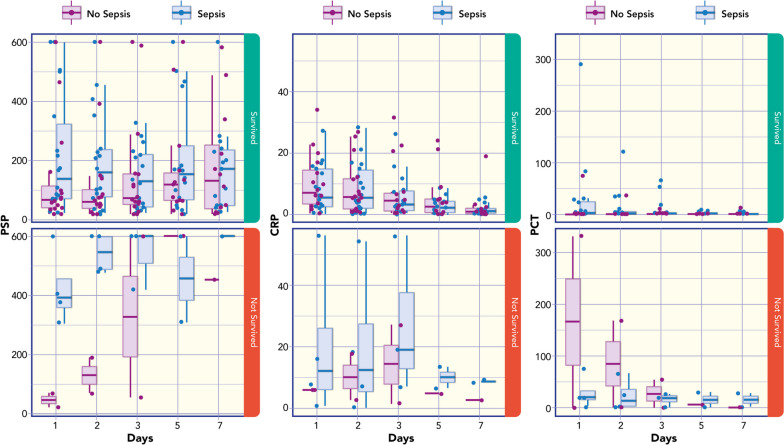
Box plots of Pancreatic Stone Protein (PSP), C-Reactive Protein (CRP) and Procalcitonin (PCT) median values at different time points in relation to the outcome (survival versus no survival)

**Fig. 4:**
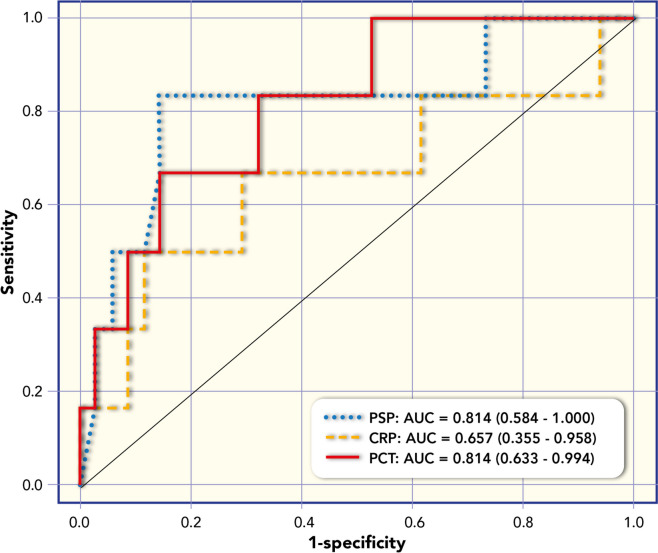
Receiving operating curve (ROC) analysis of Pancreatic Stone Protein (PSP), C-Reactive Protein (CRP) and Procalcitonin (PCT) for outcome (survival versus no survival). Area under the curve (AUC) of median values of biomarkers Pancreatic Stone Protein (PSP), C-Reactive Protein (CRP) and Procalcitonin (PCT) at all time points (day 1, 2,3,5,7)

PSP concentration was correlated with PELOD-2 score (Rho = 0.404; *p* = 0.056). Figure 3 shows box plots of PSP, CRP and PCT median values at different time points and in relation to the survival outcome. ROC analysis for survival has showed: AUC 0.814 for PSP; AUC 0.814 for PCT; AUC of 0.657 for C-RP (Fig. 4). Based on ROC analyses of all median values at different time-points the optimal cut-off for PSP to predict survival was 421.0 with a sensitivity of 83% and a specificity of 85%.

Table 5 reports the diagnostic performance of the three biomarkers (median time) in distinguishing survival from no survival in population analyzed.

**Table 5:** Diagnostic performance of biomarkers including median of five timepoints (T1 = day 1, T2 = day 2, T3 = day 3, T4 = day 5, T5 = day 7) in distinguishing survival from no survival in paediatric patients observed

Marker	AUC 95%CI	Cut-off	Sensitivity 95%CI	Specificity 95%CI	PPV 95%CI	NPV 95%CI	LR + 95%CI
PSP Median value	0.814 (0.584–1.000)	421	0.833 (0.359, 0.996)	0.853 (0.689, 0.950)	0.500 (0.187, 0.813)	0.967 (0.828, 0.999)	5.667 (2.339, 13.731)
PCR Median value	0.657 (0.355–0.958)	15.1	0.500 (0.118, 0.882)	0.882 (0.725, 0.967)	0.429 (0.099, 0.816)	0.909 (0.757, 0.981)	4.250 (1.255, 14.391)
PCT Median value	0.814 (0.633–0.994)	14.4	0.667 (0.223, 0.957)	0.853 (0.689, 0.950)	0.444 (0.137, 0.788)	0.935 (0.786, 0.992)	4.533 (1.688, 12.171)

*PSP* Pancreatic Stone Protein, *C-RP* C reactive protein, *PCT* procalcitonin, *AUC* Area Under the Curve, *CI* confidence interval, *PPV* positive predictive value, *NPV* negative predictive value, *LR* + positive likelihood ratio

## Discussion

In this pilot study, we have explored the accuracy of PSP in the diagnosis of sepsis in children and we have shown that also in pediatric setting PSP could be a promising bedside biomarker that can help clinicians to rapidly evaluate an infective etiology of systemic inflammatory response syndrome with a point of care technology.

Schlapbach et al. have described PSP normal values in different age groups in healthy children, including neonates, showing an age-dependent increase of PSP from birth to childhood, ranging from 1.0 to 16.1 ng/ml [10]. The authors in this latter study used an ELISA methodology to measure PSP [10], while we used a point of care test based on a nanofluidic technology: the two methods’ comparison showed a 4.6 fold [11].

PSP has been also investigated in neonatal sepsis showing a performance comparable or superior to other biomarkers (PCT, CRP) [12]. Furthermore, PSP has been evaluated alone or in combination with other inflammatory biomarker in critically ill children to predict outcome, showing a correlation with the illness severity and 28-day mortality [13, 14]. Our study confirm the potential role of PSP as prognostic biomarker to predict mortality in critically ill children: we have found an AUC 0.814 for PSP to distinguish survived versus no survived reporting a better performance in comparison to what was observed by Wu et al. with an AUC 0.73 [14]. In comparison with the previous study, considering the small cohort enrolled, we have not evaluated the combined AUC value of prognostic performance of PSP, PCT and CRP. Furthermore we have explored the diagnostic performance of PSP to distinguish sepsis versus non sepsis patients. The rational to measure PSP levels at different timepoints (Time 1, 2, 3, 5 and 7) in our study was based on the evaluation of PSP to early detect sepsis and its evolution in patients admitted in pediatric ward, emergency department and in pediatric intensive care, thus in patients with different severity of the sepsis’ clinical picture.

Many publications have shown in adult population PSP high accuracy in the diagnosis of sepsis and in the prediction of mortality. The currently available evidences about PSP do not allow to clearly identify a cut-off value for the early diagnosis of sepsis. A unique feature of PSP is to the early increase above the normal levels, starting before the development of clinical signs and symptoms of sepsis. In a pivotal study, serial measurements of PSP in a multiple trauma population identified in most of the enrolled patients who were developing sepsis prior to the ascent of other biomarkers and prior to clinical diagnosis [15]. Additionally, in a population of patients with sepsis-related complications, PSP levels have shown high diagnostic accuracy for discriminating peritonitis severity and predicting ICU mortality [16]. In another study, PSP levels reflected organ dysfunction and predicted death in ventilator-associated pneumonia (VAP), which allowed the stratification of patients with good and poor outcome [17]. The predictive value of PSP for in-hospital mortality in patients with severe sepsis and septic shock who require intensive care management was further demonstrated by Que et al., who also highlighted in another research that a model combining severity scores with PCT and PSP improves the prediction of mortality in these patients [18].

We have found an optimal Youden cut-off for PSP of 167 ng/ml for diagnostic accuracy of sepsis at timepoint 1 (day 1) resulting in a sensitivity of 59% but with a specificity of 83%, equal to PCT ones. For each patient, the values of the three biomarkers were highly fluctuating among the different time points potentially also influenced by antimicrobials therapies. Therefore, to have more robust data, we have summarized the five measures by the median value and, interestingly, we observed almost the same cut-offs (167 ng/ml versus 166 ng/ml) for the timepoint 1 (day 1) and the median of the five timepoints during the study time.

Previous studies have not found significant differences in PSP values in pediatric patients with infectious SIRS (sepsis) versus noninfectious SIRS, when a clinical condition of severe organ dysfunction was present [13]. Instead, we have found a higher AUC in the diagnosis of sepsis versus no-sepsis in the median of all timepoints determined for all observations, thus also in those children who showed clinical evolution with multiple organ dysfunction from the admission to the timepoint 7 (day 7). According to ROC analysis for outcome (survival versus no survival) PSP performance is equal to PCT*.* We have found an optimal PSP cut-off of 421 ng/ml to predict mortality in our pediatric population with a sensitivity of 83% and a specificity of 85%. Jiri et al. observed a cut-off value of 858 to best discriminate pediatric patients with PELOD < 12 and 12 and higher [13], but in our population PELOD-2 score median values were lower (median values 6), thus we were not able to show strong relationship between PSP values and high values of PELOD-2 score.

We have observed higher PSP values in the median of patients with sepsis and septic shock in comparison to those pediatric patients with SIRS and SIRS associated to organ dysfunction. Severe sepsis patients’ median values of PSP resulted inferior to those ones showed by patients with sepsis, we conjecture that this result could be associated to the etiology of the infection (viral infections versus bacterial infections), but further studies on larger population are required to confirm this hypothesis. Furthermore, we have to consider that the definition of severe sepsis was recently abolished in adult population with new SEPSIS-3 criteria, thus a low clinical feasibility of this categorization should be considered.

One of the strengths of our study is that this is the first time the accuracy of PSP was explored in the diagnosis of sepsis in pediatric patients admitted to the hospital with signs and symptoms of systemic inflammation associated or not with organ failure. We have also evaluated patients with multiple time points measurements during a 7 days observational period which represents an appropriate time for the evaluation of the incidence and the evolution of sepsis clinical signs. We have also showed that PSP could be used at bedside, with a Point Of Care test measurement which could be the major advantage for clinicians.

Our study has also some limits. Firstly, this is a preliminary pilot study with a need of a confirmative study in larger pediatric population. Furthermore, future studies on greater number of patients will allow to better explore the role of PSP also in pediatric population both in the diagnosis of localized infections versus sepsis and in relation to the microbiological etiology of the infection.

## Conclusions

To the best of our knowledge, this is the first study on a pediatric population about the accuracy of PSP in the diagnosis of sepsis. In our exploratory experience, in our small population, PSP could distinguish sepsis from noninfectious systemic inflammation, even if showing a relatively low sensitivity. Further studies on a larger cohorts are needed to confirm these preliminary results.

## Data Availability

All data analyzed and discussed in the framework of this study are included in this published article. The corresponding author may provide specified analyses or fully de-identified parts of the dataset upon reasonable request.

## Abbreviations

PSP: Pancreatic Stone Protein
CRP: C-Reactive Protein
PCT: Procalcitonin
PICU: Pediatric Intensive Care Unit
SIRS: Systemic Inflammation Response Syndrome
ROC: Receiver Operating Characteristic
AUC: Area Under the Curve (AUC)
